# CAST controls presynaptic sequestration of Rab6 in neurons

**DOI:** 10.1186/s13041-026-01330-1

**Published:** 2026-07-10

**Authors:** Yamato Hida, Atsushi Shimada, Yeon-Jeong Kim, Miyuki Kato-Murayama, Mikako Shirouzu, Toshihisa Ohtsuka

**Affiliations:** 1 Department of Biochemistry, Graduate School of Medicine/Faculty of Medicine, University of Yamanashi, 1110 Shimokato, Chuo, Yamanashi 409-3898 Japan; 2 Research Promotion Unit, Advanced Research Initiative, Medical Institute of Bioregulation, Kyushu University, Fukuoka, 812-8582 Japan; 3 Laboratory for Functional and Structural Biology, RIKEN Center for Integrative Medical Sciences, Yokohama-Shi, Kanagawa 230-0045 Japan

**Keywords:** Rab6, CAST, Presynaptic bouton, Rab GTPase, Membrane trafficking, Synaptic scaffold proteins

## Abstract

CAST is a core active zone scaffold protein, yet its role in Rab6-dependent trafficking remains unclear. Here, we identify the coiled-coil domain of CAST (CC10) as a direct Rab6-binding module and show that CAST selectively interacts with the GTP-bound form of Rab6 in both heterologous cells and neurons. Biochemical mapping, isothermal titration calorimetry, and bimolecular fluorescence complementation demonstrate that CC10 is necessary and sufficient for Rab6 recognition. In cultured hippocampal neurons, CAST promotes the presynaptic accumulation of Rab6, whereas CC10-disrupting mutations abolish this effect without detectably altering Rab6 distribution within axonal regions under our imaging conditions. These results define a CAST-dependent mechanism that spatially restricts Rab6 at presynaptic boutons, extending ELKS-based models of Rab6 cargo capture and providing a structural basis for the organization of presynaptic trafficking.

## Introduction

CAST (also known as ELKS2 or ERC2) and ELKS (also known as ELKS1, ERC1 or Rab6IP2) are core scaffold proteins of the presynaptic active zone that organize the molecular architecture required for neurotransmitter release [1–4]. Through their coiled-coil–mediated interactions with active zone components, including Bassoon, Piccolo, Liprin-α, and the PDZ domain–containing protein RIM1, these scaffolds link synaptic vesicle trafficking to the release machinery [5, 6]. While ELKS is broadly expressed, CAST is enriched in neurons, suggesting both shared and specialized functions between these two scaffold proteins [7, 8].

ELKS was originally identified as an activating fusion partner of the RET tyrosine kinase and subsequently identified as a Rab6-interacting protein [9, 10]. Rab6 is a small GTPase that regulates post-Golgi trafficking and polarized secretion [11–13]. In non-neuronal cells, ELKS cooperates with Rab6 to capture transport carriers at cortical secretion sites and promote vesicle docking and fusion [11]. In neurons, ELKS has been implicated in vesicle priming and in the capture of Rab6-positive carriers at presynaptic terminals, consistent with a conserved role in organizing localized secretion platforms [14–16], a process that resembles secretory hotspot formation described in non-neuronal ELKS platforms associated with LL5β [11, 16]. Structural studies have further defined the Rab6-binding region of ELKS and demonstrated direct interaction with Rab6 in vitro [14, 17].

Despite these advances, the contribution of CAST to Rab6-dependent trafficking remains unclear. Most studies have focused on ELKS, and direct evidence for CAST–Rab6 interaction in cells or neurons is lacking [17, 18]. In addition, whether Rab6 engagement by ELKS family proteins depends on its nucleotide state in cells has not been fully established. This question is particularly relevant given that Rab6A/B function is required for polarized trafficking and neuronal development, highlighting the importance of its spatial regulation [12, 19].

Previous work demonstrated that ELKS1 (ERC1) contributes to the capture of Rab6-positive transport vesicles at presynaptic terminals [14]. In contrast, the role of CAST (ELKS2/ERC2) in Rab6-dependent trafficking remains less well defined. In this study, we examine the interaction between CAST and Rab6 and its functional impact on Rab6 distribution. Using biochemical mapping, biophysical binding assays, and live-cell imaging approaches including bimolecular fluorescence complementation, we identify the tenth coiled-coil domain of CAST (CC10) as a Rab6-binding module and show that CAST preferentially associates with the GTP-bound form of Rab6 in cells. In cultured hippocampal neurons, CAST promotes presynaptic accumulation of Rab6, whereas disruption of the CC10 region abolishes this enrichment. These findings reveal a CAST-dependent mechanism that contributes to the spatial organization of Rab6 at presynaptic compartments.

## Results

### CC10 of CAST mediates direct interaction with Rab6

**Fig. 1:**
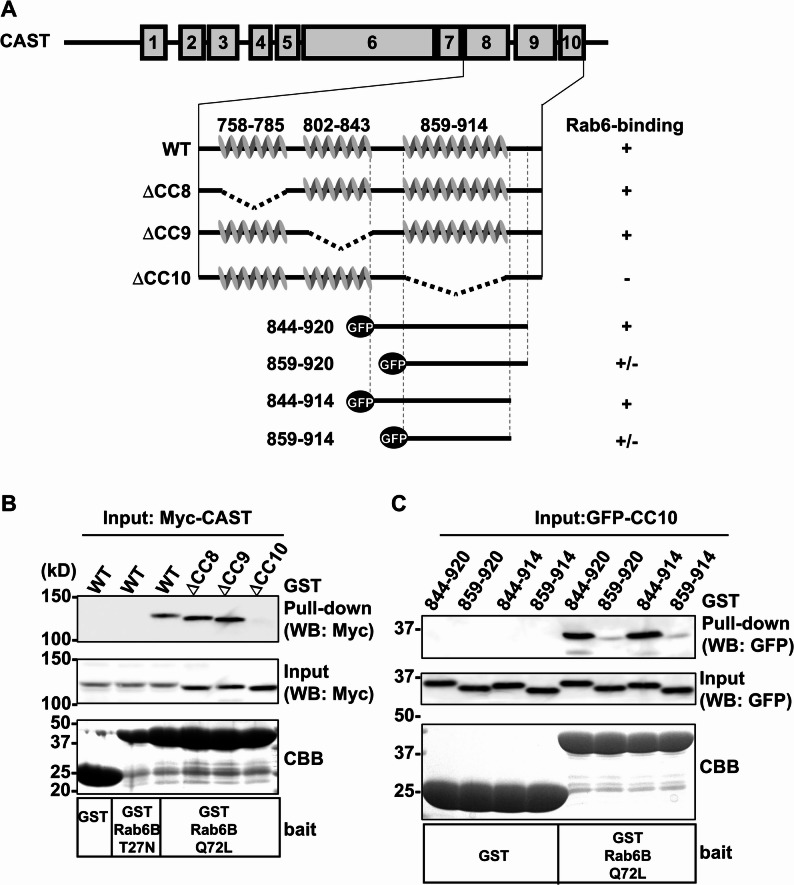
Identification of the Rab6-binding region in CAST by deletion analysis **A** Schematic representation of full-length CAST and the deletion or truncation constructs used for Rab6-binding analysis. Upper, overall domain organization of CAST. Lower, enlarged view of the C-terminal region containing CC8–CC10 and the tenth coiled-coil domain (CC10). Deleted regions or isolated fragments and Rab6-binding activity are indicated. **B** GST pull-down assay examining Rab6 binding to CAST deletion mutants. Lysates from COS-7 cells expressing Myc-tagged CAST or coiled-coil deletion mutants (ΔCC8, ΔCC9, and ΔCC10) were incubated with glutathione-Sepharose beads containing GST, GST–Rab6-GDP (T27N), or GST–Rab6-Q72L. Bound proteins were analyzed by immunoblotting with an anti-Myc antibody. **C** GST pull-down assay using the CC10 region of CAST. COS-7 cells expressing GFP-tagged CC10 were subjected to pull-down with GST or GST–Rab6-Q72L. Bound proteins were detected by immunoblotting with an anti-GFP antibody

To identify the region of CAST responsible for Rab6 interaction, we mapped the Rab6-binding site using deletion mutants targeting individual coiled-coil domains (Fig. 1A). These constructs were expressed in COS-7 cells and analyzed by glutathione S-transferase (GST) pull-down assays.

Myc-tagged full-length CAST was efficiently recovered with GST-fused constitutively active Rab6B (GST–Rab6B-Q72L), whereas no detectable binding was observed with the dominant-negative Rab6B mutant (GST–Rab6B-T27N) or GST alone (Fig. 1B). Deletion of the C-terminal coiled-coil region CC10 abolished Rab6 binding, while deletion of CC8 or CC9 reduced, but did not eliminate, the interaction (Fig. 1B). These results indicate that CC10 is critical for Rab6 association.

To determine whether CC10 is sufficient for Rab6 binding, GFP-tagged CC10 fragments were expressed in COS-7 cells and subjected to GST pull-down assays using GST–Rab6B-Q72L. The isolated CC10 fragment (residues 844–914) was efficiently recovered with Rab6B-Q72L (Fig. 1C), indicating that this region is sufficient to mediate Rab6 interaction.

**Fig. 2:**
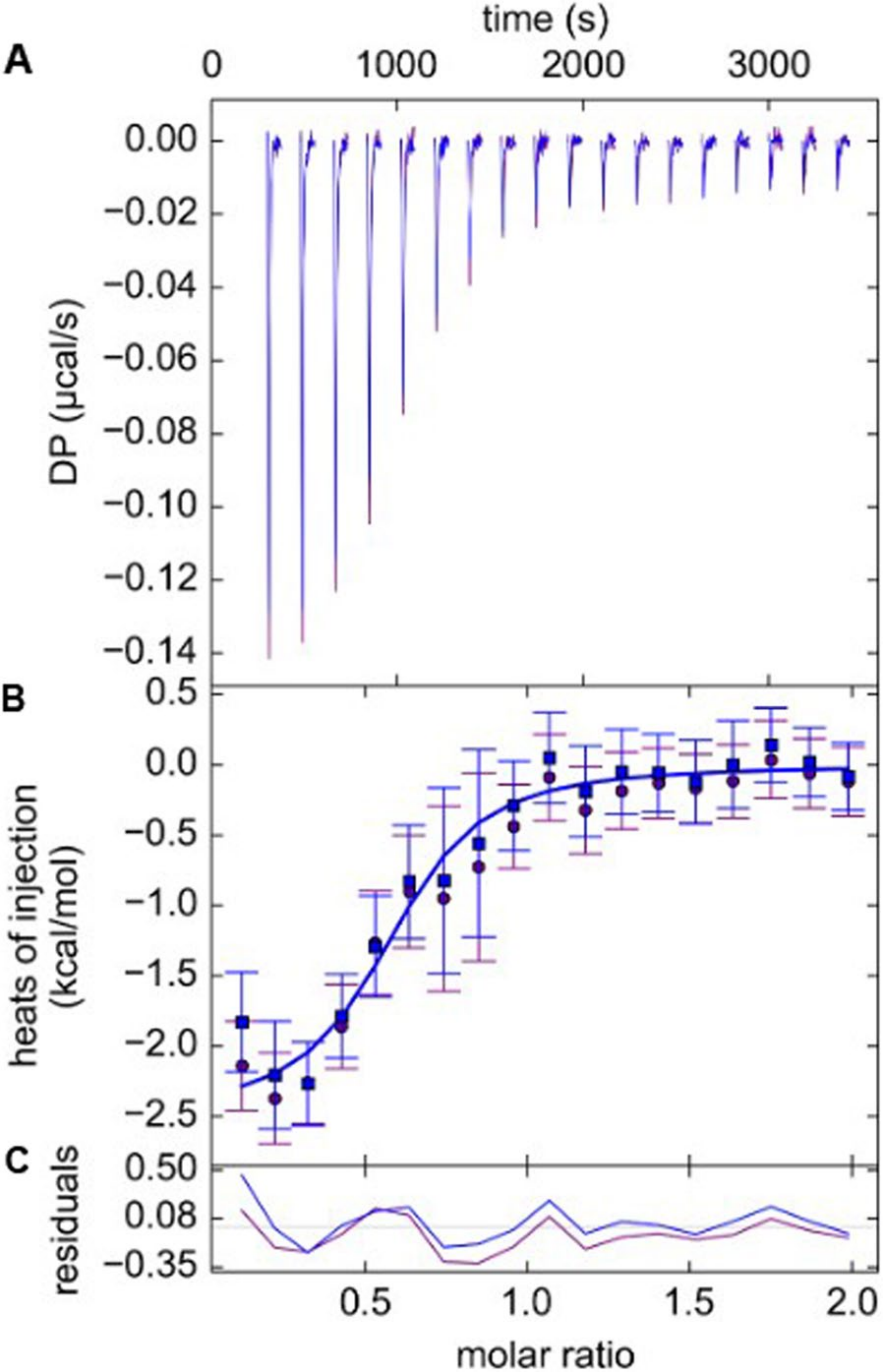
ITC of Rab6 titrated with the CC10 region of CAST **A** Baseline-corrected measured power differential (DP) as a function of time, for two independent ITC experiments (blue and red). **B** A nonlinear global weighted least-squares fit of heat released as a function of the added ligands. The errors arising from the baseline uncertainty were estimated with the program NITPIC [25], and are shown. The data were fitted with a 1:1 binding curve. **C** Residuals of the fit with a 1:1 binding curve, ranging from − 0.35 to 0.50 kcal/mol

We next tested whether this interaction is direct using purified proteins. Isothermal titration calorimetry (ITC) revealed reproducible heat changes upon injection of CC10 (residues 801–923) into Rab6B-Q72L (Fig. 2A). The resulting binding isotherms were well fitted by a single-site binding model (Fig. 2B), yielding a dissociation constant (*K*_d_) of 1.12 ± 0.58 μM and a binding stoichiometry (N) of 0.57 ± 0.05 (Table 1). The interaction was characterized by a negative enthalpy change (Δ*H* =  − 2483 ± 219 cal/mol) and a favorable entropic contribution (−TΔ*S* = − 5633 cal/mol), resulting in a negative free energy value (ΔG = − 8116 cal/mol), showing that binding is energetically favorable under these conditions. These thermodynamic parameters demonstrate that CC10 directly associates with Rab6 with micromolar affinity.

**Table 1:** *K*_d_ of CAST CC10 (801–923) with Rab6B (Q72L) determined by ITC

Protein	*K*_d_ (μM)	N	Δ*H* (cal/mol)	− TΔ*S* (cal/mol)	Δ*G* (cal/mol)
CAST CC10 (801–923)	1.12 ± 0.58	0.57 ± 0.05	− 2483 ± 219	− 5633	− 8116

N is the binding ratio of Rab6B (Q72L) to CAST CC10 (801–923).

Together, these findings identify CC10 as the principal Rab6-binding region of CAST and establish a direct interaction between CAST and Rab6.

## CAST preferentially interacts with active Rab6 in cells and axons

Having identified CC10 as a Rab6-binding region in vitro, we next examined whether CAST interacts with Rab6 in living cells and whether this interaction depends on the nucleotide state of Rab6. To visualize CAST–Rab6 interactions, we used a bimolecular fluorescence complementation (BiFC) approach [20].

**Fig. 3:**
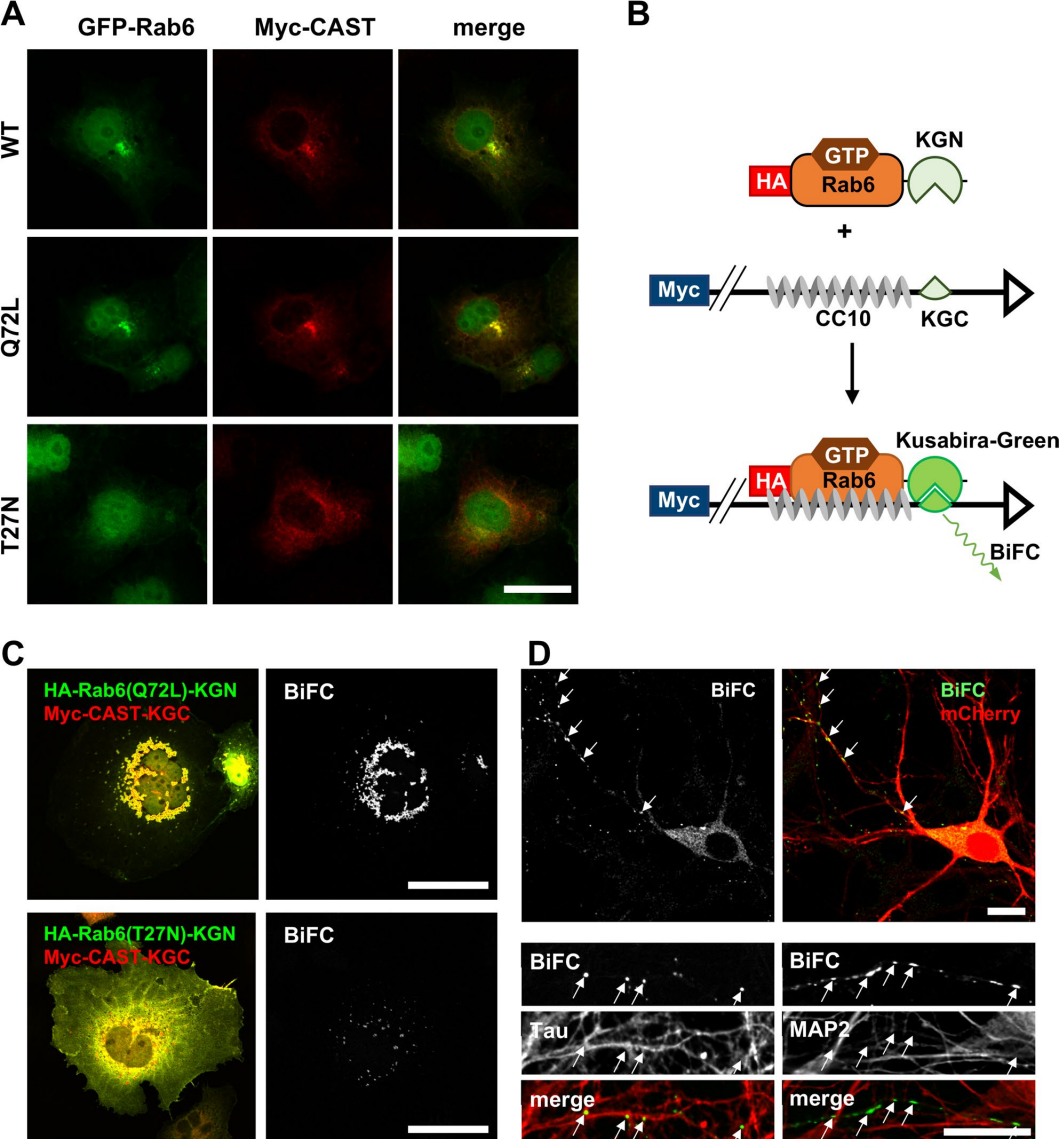
CAST preferentially interacts with the active form of Rab6 in cells and neurons **A** Subcellular localization of CAST and Rab6 in COS-7 cells. Cells expressing Myc-CAST and Rab6B-Q72L were immunostained with an anti-Myc antibody. CAST and Rab6B-Q72L show overlapping localization in the perinuclear Golgi region. Scale bar, 30 μm. **B** Schematic representation of bimolecular fluorescence complementation (BiFC) constructs. CAST was fused to the C-terminal fragment of Kusabira–Green (KGC), and Rab6 was fused to the N-terminal fragment (KGN). Interaction between the two proteins reconstitutes Kusabira–Green fluorescence. **C** BiFC analysis in COS-7 cells. Robust fluorescence was observed upon coexpression of CAST-KGC with Rab6B-Q72L-KGN, whereas minimal fluorescence was detected with Rab6B-T27N-KGN. Scale bar, 60 μm. Green signal indicates HA staining, as indicated in each panel; red signal indicates Myc staining. **D** BiFC analysis in cultured hippocampal neurons. mCherry was coexpressed as a cytoplasmic marker to visualize neuronal morphology. BiFC signals corresponding to CAST–Rab6 interactions were predominantly detected along Tau-positive axons, whereas MAP2-positive dendrites showed minimal signal. Scale bar, 30 μm (top), 20 μm (bottom)

Myc-tagged CAST colocalized with Rab6B-Q72L at the Golgi region in COS-7 cells (Fig. 3A), consistent with the known localization of active Rab6 during late secretory transport [12]. BiFC constructs were generated by fusing CAST to the C-terminal fragment of Kusabira Green (KGC) and Rab6 to the N-terminal fragment (KGN) (Fig. 3B).

Coexpression of these constructs in COS-7 cells produced robust BiFC fluorescence with Rab6B-Q72L, indicating interaction between CAST and the GTP-bound form of Rab6 (Fig. 3C). In contrast, Rab6B-T27N generated minimal fluorescence, demonstrating that CAST preferentially associates with active Rab6 in cells.

To examine the subcellular distribution of this interaction in neurons, we performed BiFC in cultured hippocampal neurons. mCherry was coexpressed to visualize neuronal morphology. BiFC signals were predominantly detected along Tau-positive axons, whereas MAP2-positive dendrites exhibited little signal (Fig. 3D).

These results indicate that CAST preferentially interacts with GTP-bound Rab6 and that this interaction is enriched within the axonal compartment.

## Mutations within CC10 reduce Rab6 association

**Fig. 4:**
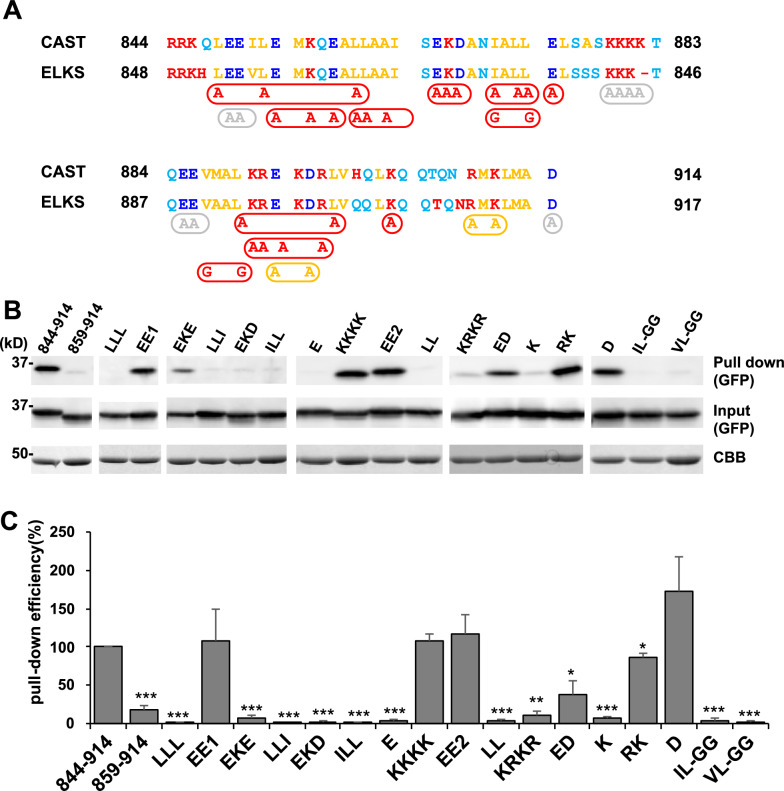
Mutations in the CC10 coiled-coil domain reduce Rab6 association **A** Alignment of amino acid sequences of the Rab6-binding coiled-coil regions in CAST (residues 844–914) and ELKS (residues 848–917). Conserved residues in CAST targeted for alanine or glycine substitution are indicated. **B** GST pull-down assay assessing the effect of CC10 mutations on Rab6 association. Lysates from COS-7 cells expressing GFP-tagged CAST CC10 (residues 844–914) or the indicated mutants were incubated with GST–Rab6B-Q72L immobilized on glutathione–Sepharose beads. Bound proteins were detected by immunoblotting with an anti-GFP antibody. **C** Quantification of pull-down efficiency for wild-type CC10 and the indicated mutants. Data represent mean values from independent experiments. Values represent the mean ± SE from independent experiments (*n* = 15 for the 844–914 and 859–914 mutants; *n* = 3 for all other mutants). Statistical significance was assessed by one-way ANOVA. * *P* < 0.05, ** *P* < 0.01, *** *P* < 0.001

To identify residues within CC10 required for Rab6 binding, we introduced alanine substitutions at conserved positions shared between CAST and ELKS within the coiled-coil region (Fig. 4A). Sequence alignment revealed several conserved residues, suggesting their potential involvement in Rab6 recognition.

GST pull-down assays using constitutively active Rab6B-Q72L as bait showed that multiple alanine substitutions markedly reduced recovery of the CC10 fragment (Fig. 4B). Quantitative analysis confirmed a significant decrease in binding compared with the wild-type CC10 fragment (Fig. 4C).

These results identify specific conserved residues within CC10 that are required for efficient Rab6 association and support the role of this coiled-coil region as a key Rab6-binding interface in CAST.

## CAST promotes Rab6 enrichment at presynaptic boutons

To determine whether CAST influences Rab6 distribution in neurons, we examined Rab6 localization in cultured hippocampal neurons expressing CAST. Neurons were co-transfected with Myc-CAST and GFP–Rab6A and stained with markers for axons (Tau), presynaptic boutons (Bassoon), and dendrites (MAP2).

**Fig. 5:**
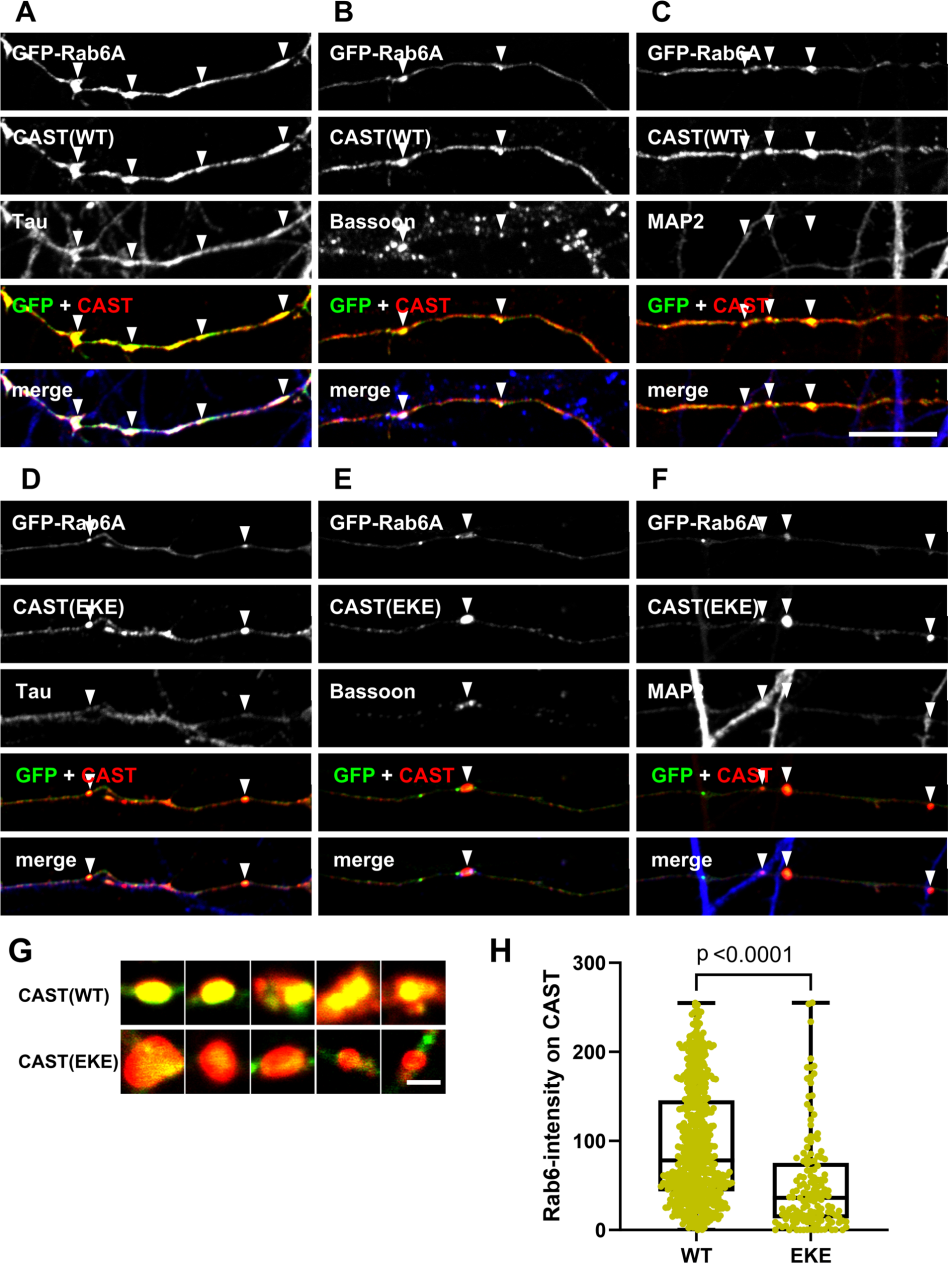
CAST promotes Rab6 enrichment at presynaptic boutons **A**–**C** Cultured hippocampal neurons coexpressing Myc-CAST (WT) and GFP-Rab6A were immunostained with an anti-Myc antibody together with anti-Tau (**A**), anti-Bassoon (**B**), or anti-MAP2 (**C**) antibodies. Scale bar, 20 μm. **D**–**F** Cultured hippocampal neurons coexpressing Myc-CAST (EKE mutant) and GFP-Rab6A were immunostained with an anti-Myc antibody together with anti-Tau (**D**), anti-Bassoon (**E**), or anti-MAP2 (**F**) antibodies. Scale bar, 20 μm. **G** Representative images showing colocalization of GFP-Rab6A and Myc-CAST at presynaptic boutons in neurons expressing wild-type or EKE mutant CAST. Scale bar, 3 μm. **H** Quantification of GFP-Rab6A fluorescence intensity at CAST-positive puncta. Data are presented as box-and-whisker plots showing the median and interquartile range, and minimum-to-maximum whiskers, with individual particles are shown as dots. WT, 564 particles from 66 cells; EKE, 145 particles from 53 cells. Statistical significance was assessed using the Mann–Whitney test

Expression of wild-type CAST resulted in prominent enrichment of GFP–Rab6A along Tau-positive axons (Fig. 5A). Within these axons, Rab6 puncta frequently colocalized with Bassoon-positive presynaptic boutons (Fig. 5B), whereas MAP2-positive dendrites showed minimal overlap (Fig. 5C). These observations indicate preferential localization of Rab6 to axonal and presynaptic compartments.

To examine whether this enrichment depends on CAST–Rab6 interaction, we analyzed a CAST mutant carrying substitutions within the CC10 region (EKE) that reduce Rab6 binding. In neurons expressing this mutant, GFP–Rab6 remained distributed along axons but exhibited reduced accumulation at Bassoon-positive boutons compared with neurons expressing wild-type CAST (Fig. 5D–F).

Representative images of Rab6 and CAST localization at presynaptic boutons (Fig. 5G) show colocalization at these sites. Quantification of GFP–Rab6 fluorescence intensity within CAST-positive boutons revealed significantly greater enrichment in neurons expressing wild-type CAST than in those expressing the EKE mutant (Fig. 5H).

Total GFP–Rab6 fluorescence and the axonal distribution of CAST were comparable between conditions, indicating that CC10-dependent interaction specifically regulates local Rab6 enrichment rather than overall Rab6 levels or CAST localization.

Together, these results demonstrate that CAST promotes the accumulation of Rab6 at presynaptic boutons and that CC10-mediated Rab6 binding is required for this spatial enrichment.

## Discussion

We identify CAST as a Rab6-interacting scaffold that promotes the accumulation of Rab6 at presynaptic boutons. CAST associates with Rab6 through a defined coiled-coil region within the CC10 domain, and disruption of this region impairs both Rab6 binding and its presynaptic enrichment. Domain mapping (Fig. 1) and biochemical reconstitution (Fig. 2) demonstrate that CC10 is both necessary and sufficient for Rab6 interaction. In addition, BiFC reveals preferential association of CAST with the GTP-bound form of Rab6 in cells and neurons (Fig. 3). Together with mutational analysis (Fig. 4) and neuronal imaging (Fig. 5), these results link a specific structural element of CAST to the spatial organization of Rab6 at presynaptic sites.

Previous biochemical and structural studies established ELKS proteins as Rab6 effectors [14, 17]. In particular, ELKS (ELKS1) and CAST (ELKS2) bind Rab6 through a C-terminal coiled-coil region with micromolar affinity, as demonstrated by isothermal titration calorimetry and structural analyses [17]. The affinity measured here for the CAST CC10–Rab6 interaction (*K*_d_ = 1.12 ± 0.58 μM; Table 1) falls within a similar range, indicating that CAST and ELKS share comparable binding strength for Rab6. This similarity supports a conserved mode of Rab6 recognition among ELKS family scaffolds. Notably, the observed binding stoichiometry (N ≈ 0.57) deviates from a simple 1:1 interaction and may reflect incomplete activity of the purified proteins or heterogeneity in the binding-competent fraction under the experimental conditions. Or due to the difficulty of assessing the precise concentration of GTP-bound Rab6B with the Bradford method, as the concentration of GTP-bound Rab6B cannot be determined based on the extinction coefficient of the protein due to the presence of GTP. Despite this, the consistent micromolar affinity and reproducible thermodynamic signature (ΔH < 0) support a specific and energetically favorable interaction.

Previous work demonstrated that ELKS (ELKS1/ERC1/Rab6IP2) contributes to the capture of Rab6-positive transport vesicles at presynaptic terminals [14]. Our findings do not suggest that CAST replaces this function. Rather, CAST represents an additional Rab6-interacting active-zone scaffold that contributes to the enrichment of Rab6 at presynaptic sites. The precise relationship between ELKS- and CAST-dependent mechanisms remains to be determined and may involve distinct, cooperative, or partially overlapping functions in the presynaptic localization of Rab6.

The mechanism by which CAST promotes presynaptic Rab6 enrichment remains unclear. Because CAST is concentrated at active zones, it is unlikely to function as a long-range recruiter of Rab6-positive carriers. Instead, CAST may act locally after Rab6-positive carriers reach presynaptic boutons by promoting the capture, retention, or enrichment of Rab6 through its interaction with active Rab6. Although the present data do not distinguish among these possibilities, they support a model in which the CAST–Rab6 interaction contributes to the spatial organization of Rab6 at presynaptic sites.

Importantly, whereas previous studies primarily relied on purified systems, our data extend these findings by demonstrating nucleotide-dependent CAST–Rab6 interaction in intact cells and neurons (Fig. 3), and by linking this interaction to Rab6 positioning at presynaptic boutons. These observations support a model in which CAST selectively recognizes the active form of Rab6 within a physiological condition. Consistent with this interpretation, wild-type Rab6, which dynamically cycles between GDP- and GTP-bound states, is expected to contain a population of active GTP-bound molecules capable of interacting with CAST.

One limitation of the present study is that the neuronal analyses relied on exogenous expression of CAST constructs. Although exogenously expressed CAST was consistently enriched at Bassoon-positive presynaptic sites, we cannot exclude the possibility that overexpression broadens its distribution beyond the endogenous active zone compartment. Therefore, the contribution of potential changes in CAST localization should be considered when interpreting the effects of exogenous CAST on Rab6 enrichment. Future studies examining the nanoscale distribution of endogenous and exogenously expressed CAST will be important for further evaluating this possibility.

The bouton-specific enrichment of Rab6 observed here (Fig. 5) is consistent with a broader model in which ELKS family scaffolds generate localized retention sites for Rab6-marked carriers. In non-neuronal cells, ELKS organizes cortical secretion platforms together with LL5β, where Rab6-positive vesicles accumulate at exocytic hotspots [11, 16]. Similar mechanisms have been described in specialized cell types, including melanocytes, where ELKS contributes to Rab6-dependent cargo targeting [21]. These findings support a conserved principle in which Rab6 marks mobile secretory carriers, while ELKS family proteins define spatial platforms that capture or retain these carriers at sites of functional demand.

Our findings further suggest that CAST contributes to this spatial organization in neurons. Rab6 is required for polarized trafficking of synaptic vesicle precursors and for normal neuronal development [14, 19]. Disruption of Rab6A/B impairs axon formation and circuit assembly, highlighting the importance of its spatial regulation [19]. The CAST-dependent enrichment of Rab6 at presynaptic boutons observed here (Fig. 5) suggests that CAST locally concentrates Rab6-associated carriers near active zones. Such positioning may enhance the efficiency of presynaptic trafficking by increasing the local availability of vesicle precursors required for sustained neurotransmitter release.

In summary, our results demonstrate that CAST directly interacts with Rab6 and promotes its presynaptic accumulation through a CC10-dependent mechanism. By combining quantitative binding analyses with cellular and neuronal observations, this study extends previous ELKS–Rab6 findings by identifying CAST as a Rab6-interacting active zone scaffold. Our findings do not suggest that CAST acts as the sole determinant of Rab6 localization at presynaptic terminals. Rather, CAST represents an additional Rab6-interacting scaffold that contributes to the enrichment of Rab6 at presynaptic sites. Together, these findings support a model in which ELKS family proteins contribute to the spatial organization of Rab6 within presynaptic boutons, thereby facilitating the trafficking processes required for efficient neurotransmission.

## Methods

### Antibodies

The following primary antibodies were used: anti-Myc (1:1000, Roche Applied Science), anti-GFP (1:1000, Thermo Fisher Scientific), anti-HA (mouse, 1:1000, Roche Applied Science), anti-MAP2 (1:500, Sigma-Aldrich), anti-Bassoon (1:500, Stressgen), and anti-Tau (1:500, Chemicon).

For immunofluorescence, Alexa Fluor 488- or Alexa Fluor 568–conjugated secondary antibodies (1:500, Invitrogen) were used. For immunoblotting, horseradish peroxidase (HRP)-conjugated secondary antibodies (1:5000, Jackson ImmunoResearch) were used.

## Plasmid construction

Coiled-coil deletion mutants of CAST (ΔCC8, ΔCC9, and ΔCC10) were generated by ligating the corresponding N-terminal and C-terminal fragments using the In-Fusion HD Cloning Kit (Clontech). DNA fragments encoding the Rab6-binding region of CAST were subcloned into the EcoRI/SalI sites of the pCAII-EGFP vector [5].

GST-Rab6B constructs containing the T27N or Q72L mutations have been described previously [22]. Site-directed mutagenesis of the Rab6-binding region of CAST was performed using the PrimeSTAR Mutagenesis Kit (Clontech).

For bimolecular fluorescence complementation (BiFC) assays, constructs encoding the N-terminal (KGN) or C-terminal (KGC) fragments of Kusabira–Green were obtained from the CoralHue™ Fluo-chase Kit and fused to the C-terminus of CAST or Rab6 expression vectors.

## GST pull-down assay

COS-7 cells were cultured in Dulbecco’s modified Eagle’s medium (DMEM) supplemented with 10% fetal bovine serum at 37 °C in a humidified incubator with 5% CO_2_. Cells were seeded at 7.5 × 10^5^ cells per 10 cm dish one day before transfection. Plasmids encoding Myc-CAST or EGFP-tagged CAST fragments were transfected using polyethyleneimine according to the manufacturer’s instructions.

Forty-eight hours after transfection, cells were harvested and lysed to prepare total cell lysates. Glutathione-Sepharose beads (20 μl packed volume) coupled with approximately 25 μg purified GST-Rab6 were incubated for 20 min at 4 °C in buffer containing 20 mM Tris–HCl (pH 7.5), 150 mM NaCl, and 2.5 mM EDTA.

The GST-Rab6 beads were then incubated with 300 μl of cell lysates in binding buffer (25 mM Tris–HCl, pH 7.5; 150 mM NaCl; 1 mM MgCl_2_; 1% Triton X-100) in the presence of 0.5 mM GTPγS for 1h at 4 °C. Beads were washed three times with wash buffer, and bound proteins were analyzed by SDS–PAGE followed by immunoblotting with anti-Myc or anti-GFP antibodies. Signals were detected using HRP-conjugated secondary antibodies and enhanced chemiluminescence, and imaged with a LAS-3000 mini imaging system (Fujifilm).

## Immunofluorecence

COS-7 cells and primary rat hippocampal neurons were prepared as described previously [23]. Cells were fixed with 4% (w/v) paraformaldehyde in phosphate-buffered saline (PBS; pH 7.4) for 20 min at room temperature.

Non-specific binding was blocked with 4% (w/v) Block Ace for 1h, followed by incubation with primary antibodies for 1h and appropriate secondary antibodies.

Fluorescent images were acquired using a confocal laser scanning microscope (Fluoview FV1200, Olympus) equipped with a 60 × oil-immersion objective. GFP–Rab6 fluorescence intensity at CAST-positive puncta was quantified using ImageJ software (NIH). CAST-positive puncta were identified by applying an intensity threshold to the Myc-CAST channel, followed by particle detection using the Analyze Particles function in ImageJ. CAST-positive puncta were selected as regions of interest, and GFP-Rab6 fluorescence intensity within each ROI was measured after background subtraction.

## ITC measurements

ITC was performed to examine direct interactions between Rab6 and the CC10 region of CAST. Rab6B-Q72L and the CAST Rab6-binding domain (CC10 region; residues 801–923) were synthesized in the cell-free system as His-tagged proteins with a TEV protease site and purified in a manner similar to that in a previous literature [24]. Proteins were purified on a HisTrap column, eluted with imidazole, cleaved with TEV protease, and further purified by cation-exchange and gel-filtration chromatography.

For ITC experiments, Rab6B-Q72L was incubated at 4 °C for 30 min in buffer containing 20 mM Tris–HCl (pH 8.0), 150 mM NaCl, 2.5 mM MgCl_2_, 5 mM DTT, 20 mM EDTA, 2.5% glycerol, and 10 mM GTP for nucleotide exchange. Subsequently, the GTP-binding state was locked by the addition of a 35 mM final concentratin of MgCl_2_. Rab6B-Q72L and the CC10 region of CAST were dialyzed into buffer containing 50 mM Tris–HCl (pH 8.0), 100 mM NaCl, and 5 mM MgCl_2_ prior to ITC measurements. The concentration of GTP-loaded Rab6B-Q72L was determined by Bradford method. ITC experiments were performed using a MicroCal Auto-iTC microcalorimeter (Malvern Panalytical). Rab6B-Q72L (30 μM) was loaded into the sample cell, and the CAST CC10 fragment (300 μM) was titrated into the cell by sequential injections. Titrations were carried out at 25 °C with constant stirring, and each injection was followed by an interval sufficient for baseline stabilization.

The ITC experiments were repeated twice and the data were processed with the program NITPIC [25]. The two data sets from each ITC experiment were analyzed by global weighted least-squares fitting with the program SEDPHAT [26]. Figures for the ITC data were created with the program GUSSI [27].

## Bimolecular fluorescence complementation assay

BiFC assays were performed in COS-7 cells transiently expressing constructs containing the N-terminal (KGN) or C-terminal (KGC) fragments of Kusabira–Green fluorescent protein. HA-tagged Rab6 fused to KGN and Myc-tagged CAST fused to KGC were introduced at equimolar ratios by electroporation using a NEPA21 electroporator (NEPA Gene).

Cells were plated onto poly-D-lysine–coated coverslips. Two days after electroporation, cells were fixed with 4% (w/v) paraformaldehyde in PBS (pH 7.4). BiFC fluorescence signals were imaged using a confocal laser scanning microscope (Fluoview FV1200, Olympus) with a 60 × oil-immersion objective.

Fluorescence intensity of BiFC signals at CAST–Rab6 puncta was quantified using ImageJ software (NIH).

## Data Availability

All data supporting the findings of this study are included in this published article. Additional raw data and materials are available from the corresponding author upon reasonable request.

## Abbreviations

BiFC: Bimolecular fluorescence complementation
CAST: Cytomatrix-associated structural protein (ELKS2)
CC: Coiled-coil domain
DMEM: Dulbecco’s modified Eagle’s medium
ELKS: Protein rich in glutamate, leucine, lysine and serine (ELKS1)
GFP: Green fluorescent protein
GST: Glutathione S-transferase
ITC: Isothermal titration calorimetry
Kd: Dissociation constant
KGN: N-terminal fragment of Kusabira–Green
KGC: C-terminal fragment of Kusabira–Green
MAP2: Microtubule-associated protein 2
PBS: Phosphate-buffered saline
Rab6: Ras-related protein Rab6
RIM1: Rab3-interacting molecule 1
